# The VidaSana Study: Recruitment Strategies for Longitudinal Assessment of Egocentric Hispanic Immigrant Networks

**DOI:** 10.3390/ijerph15122878

**Published:** 2018-12-15

**Authors:** Mariana Lopez-Owens, Kristen Starkey, Cindy Gil, Karla Armenta, Gerardo Maupomé

**Affiliations:** 1 Department of Social and Behavioral Sciences, Richard M. Fairbanks School of Public Health, Indiana University-Purdue University Indianapolis. Indianapolis, IN 46202, USA; mlopezow@imail.iu.edu (M.L.-O.); ckminatel@gmail.com (K.S.); cigil@iupui.edu (C.G.); karmenta@iupui.edu (K.A.); 2 Indiana University Network Science Institute, Bloomington, IN 46202, USA

**Keywords:** Hispanic immigration, dental care, oral health, recruitment, Mexican Americans, Central Americans, barriers to care

## Abstract

We disseminate the recruitment strategies used in the five-year VidaSana study (started in 2017) in the Midwest region of the United States, targeting recently arrived Hispanic immigrants. VidaSana aims to follow immigrants within six months of arrival for 24 months to (1) characterize features of networks (personal and community) that improve or undermine dental health; and (2) further refine methods to quantify the evolution of egocentric networks, using social network methodology. We implemented several strategies to promote and recruit potential participants into the study. We collaborate with agents serving Indiana’s Hispanic communities using three levels of visibility. The broad level includes radio advertisements, TV interviews, newspaper advertisements, and targeted Facebook advertisements. Intermediate level visibility includes posting flyers in schools, employment agencies, immigrant welcome centers, and Hispanic businesses; making announcements at church/temple and school events; tabling at community, church and school events; and a pervasive adaptation of our strategies to the requirements of our partners. Lastly, the individualized level includes direct referrals by partners through word of mouth. From the initial 13 months of recruitment (494 screened contacts and 202 recruited participants), the most successful recruitment strategies appear to be a combination of intermediate- and individual-level strategies; specifically, face-to-face recruitment at school events, direct referrals from our community partners, and tabling at community/school/church events. The current interim findings and future final findings will help guide recruitment and retention strategies for studies focused on immigrants in the current climate of heightened immigration regulations and enforcement.

## 1. Introduction

Poor oral health has been disproportionately documented in Hispanic communities, creating substantial oral health disparities (OHD). Mexican Americans (MA) and Central Americans (CA) have some of the worst oral health markers, including outcomes for caries, gingivitis or chronic periodontitis, the likelihood of having had a dental exam for oral cancer, and for using the dental care system in the preceding 12 months [1]. Additionally, undocumented immigrants face greater barriers that prevent them from accessing government-funded health programs, such as Medicare, Medicaid or other programs that may provide dental care coverage [2]. An important first step in addressing the OHD affecting MAs and CAs is to characterize the factors and mechanisms that lead to better or worse oral health outcomes. Up-to-date descriptors of such trends is of special importance in this day and age, as more finely grained features uniting or differentiating oral health characteristics become apparent among Hispanic sub-groups [3,4,5]. To identify the differences within Hispanic subgroups, we have resorted to a new(er) paradigm in OHD research, network methods. We have found that the finely layered sets of influences within and across groups afford a more sophisticated interpretation of how personal and community networks support or undermine social norms and oral health behaviors—after taking into account common demographic variables and larger structural dimensions [6,7,8,9]. However, much remains to be established about the mechanics underlying the transactions within personal and community networks, in particular in the context of such an important network-altering event such as migration. Besides the immigration health issues with substantive implications for health outcomes, health policy, and personal well-being (e.g., dietary changes), network methods afford a unique opportunity to map over time changes in personal and community networks upon immigration.

To address this knowledge gap, we started the VidaSana study to investigate oral health disparities and overall well-being of MA and CA immigrant populations from Mexico, El Salvador, Guatemala, and Honduras residing in the Midwest region of the United States. This longitudinal study, funded by the National Institutes of Health, is mainly focused on interviewing 280 immigrants within six months of their arrival for a baseline interview—and conducting follow-up interviews at 6, 12 and 24 months. The reason for the six-month window is to identify social networks as soon as they are ‘re-starting’ after migration; simply put, a person who has (largely) severed ties with the personal networks upon a life-changing event such as migration is starting to re-create all manners of personal networks. This is a unique opportunity to map networks and their evolution. While challenging from a recruitment perspective, one major, empirically important feature of the study is that it will allow us to better quantify (and refine the analytic methods about) the evolution of egocentric networks after a major life event.

The VidaSana study has three major aims. First, to characterize the norms, attitudes, behaviors, and perceptions of barriers around dental care and oral health among recent Hispanic immigrants, with specific attention to associations between these factors and personal network characteristics. We are collecting information using survey items largely from existing national surveys, adapted through extensive formative research. Second, to determine relationships between acculturation, personal and community network dynamics, diffusion of norms and resources, and changing oral health behaviors, attitudes, and outcomes over time among MAs and CAs. We employ egocentric social network methods, in which the person being interviewed (‘ego’) will report information about him/herself, but also about the peers that make up his/her personal network (‘alters’). By quantifying the functions, closeness, and dimensions that alter the living environment of the ego, we can examine the social norms and impacts that improve or undermine health outcomes for the ego—net of individual level features such as income, education, and so on. The last aim of VidaSana is to use the longitudinal perspective accrued by extensive follow up of the immigrants to refine analytic methods to precisely quantify the evolution of networks.

The present paper illustrates several methods used for recruitment (and retention) for the ongoing VidaSana study and discusses opportunities and challenges going forward.

## 2. Methods

This study has been approved by the Indiana University IRB (#1703740862) and ensures a high level of data protection through a Certificate of Confidentiality issued by the funding agency. Study participants receive a gift card of US$50 for each interview.

For the sake of simplicity, we use the term “Hispanic” to connote our study population’s broader category, indicating that we limit our research to four nationalities of origin: Guatemalan, Honduran, Salvadorian, and Mexican.

### 2.1. Community Context

**Figure A1:**
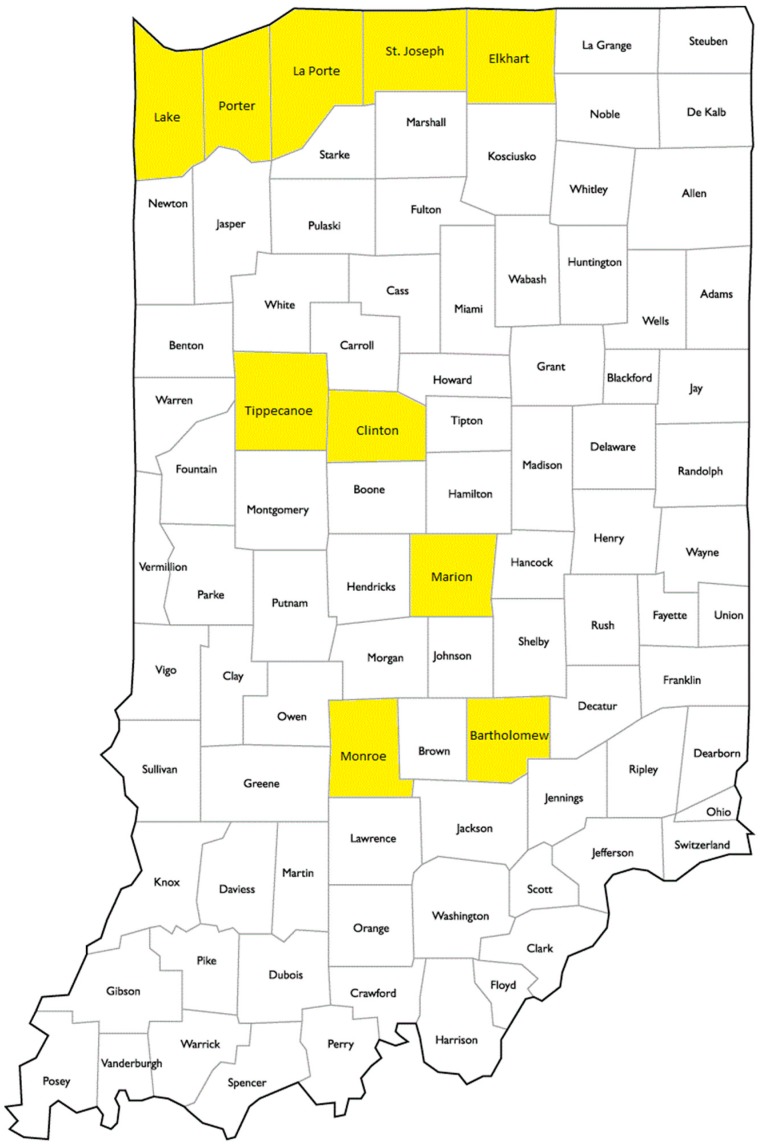
Recruitment counties for VidaSana in Indiana.

The study takes place in the state of Indiana, in the Midwest region of the United States. Recruitment began in August 2017. According to the U.S. Census, in 2017 Hispanics made up 6.8% of the population in Indiana [10]. Our recruitment efforts take place in several Indiana counties, where the number of immigrants who entered Indiana in 2010 or later was greater than in other state counties [11]. For the first eight months of recruitment, Marion County was our primary site of recruitment. Our team expanded to include Northwest Indiana in late December 2017. The inclusion of Northwest Indiana added Lake, Porter, LaPorte, St. Joseph, and Elkhart counties to our recruitment efforts, where the number of recently arrived immigrants was also higher than average for the state of Indiana in 2010. In addition, smaller operations were activated in Clinton, Monroe and Bartholomew counties. A map of the selected counties and their locations is found in Appendix A (Figure A1). In addition, Indiana ranks as the 21^st^ most populated state in the U.S. for people who identify as Hispanic or Latino/a: over 400,000 residents [12]. Besides these demographic trends, the Midwest contrasts markedly with other United States regions that have a long-standing high, Hispanic population density. This is an empirically important challenge leading to a gap in knowledge: the Midwest is one of the fastest expanding gateway locations for Hispanic immigration in recent years, [13,14]. Most large scale studies on Hispanic health have been conducted in other United States regions, such as the Southwest and West regions, with long-standing high Hispanic population density [3,4,5].

### 2.2. Recruitment

The enrollment goal of VidaSana is to recruit 560 participants; 280 participants from Mexico, El Salvador, Guatemala, or Honduras who have lived in the United States for six months or less (Focal Respondents, FR). In addition, we will utilize the base of 280 recent arrivals’ personal networks to recruit an additional 280 participants who have lived in Indiana two or more years (Network Respondents, NR).

To enroll the needed participants into our study, we created a recruitment system that involves collaborating with a variety of agents serving Indiana’s Hispanic communities. We followed common recruitment strategies reported in the literature, but our specific strategies grew out of formative research and our previous research experience in the area [15,16,17,18,19,20,21]. Every single component of the recruitment system was prepared to be conducted in Spanish; the idioms, slang and lay dental terminology have been adapted during extensive formative research to ensure currency and comprehension to the cultural contexts of the four nationalities of origin [18]. All survey materials were translated and back-translated into English and Spanish. Although materials in English are available throughout the baseline interview and the three follow up contacts, to date we have not used the English versions; each participant is given the choice of which language is employed in the interview.

Our recruitment strategies utilize three levels of visibility for potential participants (Table 1). The broadest level of visibility includes Hispanic radio advertisements, Hispanic TV interviews, Hispanic newspapers advertisements, Hispanic magazine advertisements, and disseminating targeted Facebook advertisements.

The intermediate level of visibility includes three main components: posting flyers at various locations and brochures offered throughout Central Indiana in employment agencies, immigrant welcome centers, and Hispanic businesses. Beyond disseminating study literature, intermediate level visibility includes making announcements at church/temple events; tabling at community events; and adapting our recruitment strategies to the requirements of our partners (e.g., schools and community organizations). Most of these events take place during weekends or weekday evenings.

Lastly, the individualized level of visibility includes direct referrals by community partners through word of mouth. The cross-cutting dimensions of the three levels are described below.

#### 2.2.1. Phone/Texting System

A telephone system was created so that participants can reach a Spanish-speaking VidaSana staff member almost 24 h a day, 7 days a week. We have a landline phone set up at our headquarter office that participants call. If the call is not picked up, it is then forwarded to our project manager’s and Principle Investigator’s cell phone. If the call is sent to voicemail, staff return the call within 24 h.

#### 2.2.2. Activating Community Organizations

Before initiating relationships with community organizations and leaders, we prioritized Hispanic dense areas in Indianapolis where we could recruit. Study staff has been meeting with 97 community leaders (until September 2018, and more to be added); we lead those meetings with a standardized description of the opportunity for participation in the study and a culturally tailored invitation to gain the community leader’s input on different aspects of our project. We found that asking established community partners for help connecting us to other interested stakeholders allowed us to reach multiple organizations that were willing to disseminate our study information.

Through this method, we activated 141 community partners and organizations, which include non-profit, private, and public organizations in several counties throughout Indiana (Appendix A). These include employment staffing firms, health care centers, law firms, apartment complexes, mobile home communities, libraries, Hispanic retail centers, food pantries, schools, English as a Second Language (ESL) classes, and other organizations that serve Hispanics. We distributed posters, business cards, and flyers describing the study to each of our partner organizations, who would then in turn disseminate these materials to their stakeholders.

#### 2.2.3. Scheduling

The VidaSana study staff laid out an overall strategy for scheduling interviews early in the study. Nevertheless, some of the interview scheduling varies on a case-by-case basis. When the participant is interested in joining our study, they contact study staff and are screened for eligibility. Data are collected on how participants learned about our study. After a study staff member verifies that the participant is eligible, she asks about their availability. The participant is subsequently put in touch with an interviewer to schedule the administration of the survey; interviewers are chosen from a trained group of interviewers, who offer multiple options in terms of location and schedule availability. In cases where the interview is scheduled a few days out from the initial contact, the study staff reminds the interviewer and the participant of the interview the day before and a few hours before, to confirm the interview has been scheduled and it is happening. We found that contacting the participant more than two times in a row was sometimes perceived as suspicious by the participant; we adjusted our approach accordingly.

#### 2.2.4. Retention

We use various methods to retain our participants throughout the two years of follow-up in the study. The project managers send out reminder cards in Spanish to all participants at 3, 5, 9, 11, 16, 20 and 24 months. We also send birth month cards to the participants (omitting day and year in card). We are keenly aware that this is a highly migrant target population. If we cannot confirm the exact current address of the participant, we send them a text, call them, or send them a Facebook message to ensure they remember they are still a part of the study. Marketing efforts and outreach efforts help retain participants; for example, announcements are frequently made at churches and temples to keep the study visible to the community. Community partners also support the research team in reminding participants of the VidaSana study.

### 2.3. Marketing

#### 2.3.1. Brand Recognition

During the formative phase of the study, we created stationery (flyers, business cards, posters, and brochures), a study website, and a Facebook page, to cultivate an identity for our study. We based this rationale on prior experiences [22]. The posters, flyers, and brochures include a telephone/texting number, a Facebook page, and a website address that interested individuals can access to receive more information about the study.

Within the first months of recruitment, we utilized radio ads in local Spanish radio stations to introduce and familiarize our study to the community at large. Furthermore, we included study ads in local newspapers published in Spanish. Lastly, we used targeted advertising on the social networking site Facebook to recruit study participants. We utilized these broad recruitment methods on a weekly and biweekly basis. The scripts we used in the radio ads were discussed with station executives and edited in several iterations by the study team; our goals were to make them attractive and brief. Our advertisement agreement with the local Spanish radio stations included periodic interviews/appearances of the PI and project managers in Hispanic TV broadcasts, online TV shows, and radio interviews to increase visibility.

We had to think of unconventional ways to recruit participants. For example, outreach in factories. We collaborated with staffing firms who helped us to come to manufacturing factories during workers’ lunch breaks. Our staff had the opportunity to make short presentations about our study and recruit qualifying participants. In addition, we created a partnership with a large open-air style Hispanic market, the Indiana Discount Mall. In addition to including our flyers in their monthly magazine, we are able to set up an informational booth and do in-person recruitment. The VidaSana team handed out an average of 200+ flyers each time we were at the market.

Another example is using Google Maps to identify Hispanic grocery stores and businesses in areas with a high-density Hispanic population. Study staff then split up sections on the map and strategized to post VidaSana flyers in all of these businesses. Most of the Hispanic businesses have public bulletins that make it easy to post informational flyers. We found that posting flyers at grocery stores is a good opportunity for one of our bilingual staff members to speak to people one-on-one about the study so they become aware of the opportunities to participate.

In addition, we post study information within the stationery and forms distributed in the waiting rooms of different immigrant welcome centers, libraries, food shelters, and in offices, such as the Mexican Consulate in Indianapolis. Since there are no consulates in Indiana for Guatemala, El Salvador nor Honduras, we were unable to pursue those options.

#### 2.3.2. Website

The website provides information about the study’s purpose and requirements, along with endorsements from highly-visible Hispanic community organizations, media, and faith-based groups. Individuals interested in participating in the study can fill out a brief form on the website indicating their interest, and study staff member then contacts that individual to answer further questions.

### 2.4. Iterative Review of Strategies

The VidaSana study staff meets on a weekly basis. During these meetings, our team discusses current recruitment strategies, current accrual of new participants, retention challenges, and other participant specific issues, to name a few. We discuss what seems to work, what does not, and how to improve for the future. We use this time to brainstorm and examine what the next best steps would be for recruitment, and to support retention, in a constant process of evaluation and improvement. Documentation of such processes is updated on a weekly basis. We based this rationale on prior experiences [16]. The type of questions we pose for formal discussion and scheduled follow-up include: What type of organizations should we look at? How should we go about contacting organizations of interest? How useful will contacting this organization be for us, how much leverage will we get from them? We also examine trends of recruitment and retention numbers per site against calendar expectations to meet recruitment and retention targets.

## 3. Results

Between August 2017 and September 2018, we designed and implemented 15 components of our recruitment strategy (Table 1). Through this approach, 494 potential participants contacted the VidaSana study staff and 202 (40.9%) have been recruited into the study for the baseline interview. The accrual from various components of the study visibility levels is in Table 2 (depicting both screened contacts and qualifying participants). It is important to note, however, that although some contacts may have qualified for the study, 12 chose to not participate for reasons pertaining to conflicting schedules, available time to complete the survey, or upon learning that the study expected them to complete four surveys in 24 months.

## 4. Discussion

Discussion of the methods used in the VidaSana study will be limited to critically reflecting on their relative value for recruitment and retention success. At present, the study is ongoing and no interim results on quantitative outcomes are available.

### 4.1. Community Context

One dominant element in the study is that our recruitment efforts have taken place during a highly politicized time with regard to immigration. While not new, the largely adverse climate causes some recently-arrived immigrants to be hesitant to disclose personal information [23]. We have also found, from speaking to local community leaders (e.g., priests, social workers, ESL teachers), that there is considerable mistrust in the community toward many agencies. While research studies have not been singled out as an intrinsic instrument of law enforcement agencies, in the opinion of local leaders, varying levels of mistrust have increased generally across the board. They also feel that the immigration surveillance measures by the federal government have transformed public space into areas often perceived to pose heightened risks. As a consequence, immigrants (documented or not) have become less active in a range of sociocultural activities, such as going to church or walking their child to school [24,25]. These factors colored the context of our study. As highlighted previously [16,26,27], we were aware of the need to build trust continuously by immersing the research team in the living spaces of the community members.

### 4.2. Recruitment

#### 4.2.1. Phone/Texting System and Activating Community Organizations

Upholding the importance of engaging the community in the development of our outreach plan [23,28,29], we preserved a fluid interaction with the community. We contacted active leaders in the community who had been instrumental in helping our previous studies gain momentum. Recruitment during a turbulent political climate was slow when we first began; we enrolled and interviewed on average nine participants per month from September 2017–February 2018. From March 2018 to September 2018, we enrolled and interviewed on average 20 participants per month. Thus, cultivating relationships with community partners took place for several months before we began seeing enrollment numbers for the study go up.

We developed close ties with community organizations by exchanging valuable information about other resources that would be of interest to their Hispanic clientele. For example, where to find affordable English language classes. Thus, it is important that our team continues to be a part of the community and to stay up to date on the many different resources available. By providing our partners with such information, we were able to cultivate trust, and encourage mutual help. Another way VidaSana staff further developed strong ties with community organizations is by volunteering some of their time to organizations with special projects. A list of the multiple ways in which the VidaSana research team is visible and active in the community is given in Table 1.

One of the most important components in gaining the community’s trust was to hire staff members who had a high level of community involvement [26,27]. All of our study’s interviewers are bilingual, most are women, and all are heavily involved in the community (e.g., as freelance hospital interpreters). Such profiles were designed to purposely help enrollment. As suggested in prior reports [18], interviewers were extensively trained upon starting their roles, subsequently calibrated at regular intervals, and offered formative feedback when periodically observed by management staff during data collection contacts.

When we began activating schools, we utilized a top-bottom approach and began to activate partners by reaching out to school leaders. However, we found out that it was often more effective to begin to reach out to social workers and parent-teacher liaisons who work with early English learners. The school workers who work closest with the families are important in sharing our information with the families they serve. Educators are highly esteemed in the Hispanic community, playing a particular influential role in the lives of students and their families [23]. Having teachers introduce our project to their families helped us gain trust by the community.

#### 4.2.2. Scheduling and Notions about Retention

We utilize several different retention strategies with our participants. We based this rationale on prior experiences [30,31,32,33]. These include using texts, phone calls, Facebook messages, WhatsApp messages, a landline texting application that enables us to text participants from our landline, and reminder cards at 3, 5, 11, 16, and 24 months. In addition, we support higher retention rates by assigning the same interviewer for the duration of the study—to the extent it is feasible. Such strategy helps develop strong interviewer-participant relationships over the two-year follow-up period. Recruiters, interviewers, and program staff engage participants with respect to building trust, a quality of interaction that has been shown to be key to successful retention with vulnerable Hispanic families. [26]. Although many of these retention strategies have been helpful, our team has not gone without encountering challenges that already reported in the literature when working with hard-to-reach participants [19]. Five examples of those challenges are as follows. We emphasized that our interviewers contact the participants only once or twice when trying to schedule participants for their interviews. We also learned that some of our participants did not answer calls from unrecognizable phone numbers. We learned that the study staff member that first had contact with a participant to be very clear that an interviewer would be reaching out to them; otherwise too many attempted contacts could push the participant away. Such pre-scheduling call is key to building a relationship of trust with participants during the data collection process and throughout the study. In addition, at times we perceived a participant was not answering our phone calls; in some instances, we assumed that the participant did not want to be in the study any longer. However, after holding off contacting that participant for a month, and then trying again, we found that their number was temporarily turned off, or they changed numbers, because they were not able to make a cell phone payment on time.

Certain characteristics of our target population that bear upon retention performance include some participants being relatively transient, many have unstable street addresses or phone numbers, or some being unable to speak English with enough fluency. In addition, due to low socioeconomic status for some, we have encountered that a participant was unable to pay his phone bill, causing the cell phone number to be cut and us losing touch with that participant. Our team discussed the extreme importance of having multiple forms of communication [27] (WhatsApp, Facebook, text, following up with an organization participants are associated with) to better follow the participant for the duration of the study. Multiple forms of communication were critical, e.g., when we found that a participant had to relocate following a job opportunity. In addition, we used Facebook as a search engine to find a few participants that had relocated, who would otherwise be lost to follow-up. Participants who relocated are still tracked and interviewed using long distance telephone calls and response cards uploaded on the study website; the actual procedure for data collection in follow-up sessions therefore remains largely identical to a face-to-face contact (interviewer reading the question and entering the chosen response on a tablet).

We have found that it is imperative to make the study time and scheduling requirements clear to the participants, just as reported in various studies [17,18,23,27]. At first, most participants are eager to volunteer to be part of the study; but upon learning about the two-year, four-interview requirement for the longitudinal study, a minority of participants hesitate. When participants’ follow-up interviews are due, we found that getting a hold of participants who hesitated at first when being screened, were those participants that were hard to reach in later contacts.

When scheduling interviews, the VidaSana team has been very flexible in accommodating the participants’ needs. For example, some participants have trouble finding transportation, which was an important consideration when scheduling interviews. As recommended, data collection strategies to engage Hispanic immigrant families suggest considering the lives and schedules of participants [23]. A VidaSana team member develops a relationship with each participant to be a liaison between the participant and the interviewer. This typically makes the participant feel more comfortable about our study and more willing to participate [17].

### 4.3. Corollary

The present narrative illustrates the numerous approaches and complementary ideas used by the VidaSana staff during the recruitment and retention of recently arrived Hispanic immigrants into a longitudinal study in the Midwest region of the United States. In this day and age, in which trying circumstances pose very challenging conditions to conducting health research with immigrant and minority populations, the VidaSana study has continued to succeed not only in recruiting but, most importantly, in retaining a large group of vulnerable study participants. We have worked closely and consistently with this community and the various organizations and agencies around which these families gather, striving to obtain excellent quality data to understand in accurate detail immigrant and minority health phenomena. We aim to use the data to help articulate in the future more appropriate programs and policies.

## Figures and Tables

**Table 1:** Levels of Visibility and Components for VidaSana Study.

**Broad Level of Visibility**		
**Theme**	**Example**	**Quantity**
Media Partnerships	Advertised on 19 different media outlets including radio stations, local Spanish TV news stations, three web-based Spanish Facebook shows, magazines, and several Hispanic newspapers throughout the state of Indiana.	19 media outlets
**Intermediate Level of Visibility**		
**Theme**	**Example**	**Quantity**
Employers	Discussed strategies with 3 large staffing firms. Spoke with recruiting managers of local staffing firms to get an idea of how many Hispanic workers they employed. Asked permission to distribute our flyers to their workers’ table during worker’s lunch breaks, or keep our flyers posted on their informational bulletin.	4 employers
Hispanic Retail Stores	Affixed flyers at over 100 Hispanic retail stores in Indianapolis and South Bend.	130 retail stores
Health Centers	Discussed strategies with 2 large health corporations and several clinics. Distributed flyers in waiting rooms at five primary medical care clinics with a large Hispanic population. None of these had dental care services.	7 primary care clinics
Law Firms and Immigration Advocacy Groups	Received support and advice from 3 local immigration advocacy groups regarding current migration trends. Four law firms agreed to share our information in their waiting rooms	3 local Indianapolis law firms
Housing	Distributed flyers to 12 different mobile home and apartment complex communities. Set up an outreach booth at a high-density Hispanic apartment complex, serving more than 2000 Hispanic in Central Indiana.	3 mobile home complexes and 9 apartment complexes
Libraries	Distributed flyers at 6 different Indianapolis libraries.	6 different Indianapolis libraries
Food Pantries	Distributed flyers and set up recruitment tables at over 9 different food pantry locations serving high-density Hispanic areas.	9 different food pantry locations
**Intermediate and Individualized Level of Visibility**		
**Theme**	**Example**	**Quantity**
Tabling at community events	Tabled at 50 community outreach fairs in South Bend, Indianapolis, Lafayette, and Columbus, Indiana.	50 tabling events
Adult English Classes	Made short presentations and distributed flyers to 17 different ESL adult education classes.	17 different English adult classes
School Townships	Partnered with 7 Indiana school townships. Initiatives included allowing us to table at district-wide ESL events, sharing our flyers to parents, and allowing us to post flyers on school resource bulletins.	13 different schools
Church/Temples	Partnered with 21 Indiana churches/temples. Met with faith leaders to ask permission to make announcements after religious services and hand out outreach materials afterwards.	21 churches/temples
Volunteer at Community Events	Sought organizations that assist immigrant and refugee families find community organizations and services. Volunteer activities could include helping newcomers find resources like housing, food, legal assistance, English classes, translation and transportation.	Volunteered an average of 3 h a week
Community Organizations	Partnered with 33 community organizations serving Hispanics, including Immigrant Welcome Centers, Neighborhood Community Centers, educational non-profits, anti-sexual violence non-profits, adult English class centers, children advocate groups, and other social service provides aiding Hispanic communities.	33 community center and non-profit agencies
**Individualized Level of Visibility**		
**Theme**	**Example**	**Quantity**
Direct Referrals	Direct referrals by our partners and self-referrals through our website submission form.	91 direct referrals

**Table 2:** Screening and Recruitment August 2017–September 2018.

**Total: How Participants Found out about VidaSana**	
Church locations	56
Facebook	46
Grocery store locations	21
Direct referral from partners	91
School tabling event	35
Radio advertisement	5
Clinic / Hospital locations	5
Community tabling event	111
Texted us via Zipwhip	7
VidaSana website submission form	22
Library locations	3
Food Pantry locations	38
Factory locations	2
Google advertisement	4
Newspaper advertisement	4
Magazine advertisement	7
Word of mouth	37
Sum of all contacts screened	494
**Countries of Origin for Screened Contacts**	
El Salvador	26
Honduras	33
Guatemala	19
Mexico	377
Other country	39
Total screened	494
**Countries of Origin for Qualifying Participants**	
El Salvador	19
Honduras	25
Guatemala	16
Mexico	96
Total qualifying	202
Percentage of screened vs. qualified	40.9%

## Appendix A

Shaded counties represent counties where VidaSana staff has recruited participants via at least one of the three visibility methods.
